# Coumarin/β-Cyclodextrin Inclusion Complexes Promote Acceleration and Improvement of Wound Healing

**DOI:** 10.1021/acsami.4c05069

**Published:** 2024-06-07

**Authors:** Flávia Viana Avelar Dutra, Carla Santana Francisco, Bruna Carneiro Pires, Marcella Matos Cordeiro Borges, Ana Luiza Horta Torres, Vivian Alexandra Resende, Marcella Fernandes Mano Mateus, Daniel Fernandes Cipriano, Flávio Bastos Miguez, Jair Carlos Checon de Freitas, Jéssika Poliana Teixeira, Warley de Souza Borges, Luciana Guimarães, Elaine Fontes Ferreira da Cunha, Teodorico de Castro Ramalho, Clebio Soares Nascimento, Frederico Barros De Sousa, Raquel Alves Costa, Valdemar Lacerda, Keyller Bastos Borges

**Affiliations:** † Departamento de Ciências Naturais, Universidade Federal de São João del-Rei, Campus Dom Bosco, Praça Dom Helvécio 74, Fábricas, 36301-160 São João del-Rei, Minas Gerais, Brazil; ‡ Departamento de Química, Universidade Federal do Espírito Santo, Centro de Ciências Exatas, Avenida Fernando Ferrari, S/N, Goiabeiras, 29060-900 Vitoria, Espírito Santo, Brazil; § Departamento de Medicina, Universidade Federal de São João del-Rei, Campus Dom Bosco, Praça Dom Helvécio 74, Fábricas, 36301-160 São João del-Rei, Minas Gerais, Brazil; ∥ Departamento de Física, Universidade Federal do Espírito Santo, Centro de Ciências Exatas, Avenida Fernando Ferrari, S/N, Goiabeiras, 29060-900 Vitoria, Espírito Santo, Brazil; ⊥ Instituto de Física e Química, Universidade Federal de Itajubá, 37500-903 Itajubá, Minas Gerais, Brazil; # Departamento de Química, Universidade Federal de Lavras, Campus Universitário, 37200-900 Lavras, Minas Gerais, Brazil

**Keywords:** inclusion complex, cyclodextrin, coumarin, healing wounds, skin lesions

## Abstract

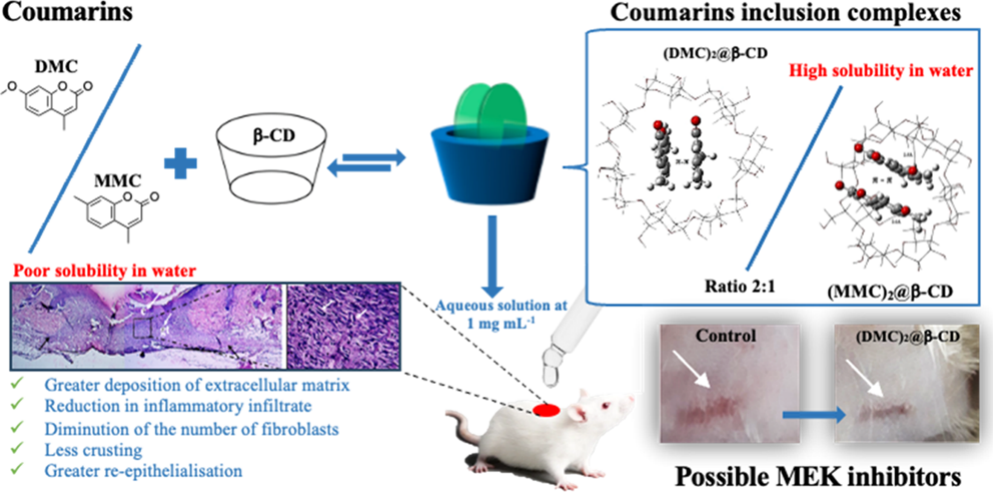

Coumarins have great pharmacotherapeutic potential, presenting several biological and pharmaceutical applications, like antibiotic, fungicidal, anti-inflammatory, anticancer, anti-HIV, and healing activities, among others. These molecules are practically insoluble in water, and for biological applications, it became necessary to complex them with cyclodextrins (CDs), which influence their bioavailability in the target organism. In this work, we studied two coumarins, and it was possible to conclude that there were structural differences between 4,7-dimethyl-2*H*-chromen-2-one (DMC) and 7-methoxy-4-methyl-2*H*-chromen-2-one (MMC)/β-CD that were solubilized in ethanol, frozen, and lyophilized (FL) and the mechanical mixtures (MM). In addition, the inclusion complex formation improved the solubility of DMC and MMC in an aqueous medium. According to the data, the inclusion complexes were formed and are more stable at a molar ratio of 2:1 coumarin/β-CD, and hydrogen bonds along with π–π stacking interactions are responsible for the better stability, especially for (MMC)_2_@β-CD. *In vivo* wound healing studies in mice showed faster re-epithelialization and the best deposition of collagen with the (DMC)_2_@β-CD (FL) and (MMC)_2_@β-CD (FL) inclusion complexes, demonstrating clearly that they have potential in wound repair. Therefore, (DMC)_2_@β-CD (FL) deserves great attention because it presented excellent results, reducing the granulation tissue and mast cell density and improving collagen remodeling. Finally, the protein binding studies suggested that the anti-inflammatory activities might exert their biological function through the inhibition of MEK, providing the possibility of development of new MEK inhibitors.

## 1 Introduction

Wound care is an established field in medicine, yet treatment of nonhealing and chronic diabetic wounds still remains a tremendous challenge. Diabetic patients face a significantly higher number of amputations, almost 10–20 times higher than nondiabetic patients.^1^ Diabetic patients (about 25%) can develop foot ulcers, leading to amputations and resulting in lifelong disabilities.^2,3^

Skin healing remains a challenge due to the complex factors involved in tissue injury,^4^ which can lead to a well-orchestrated inflammatory response. Immediately after cutaneous wounding, the clotting cascade forms a fibrin clot that stops blood loss and initiates the rescue of tissue integrity.^5^ Then, the inflammatory phase begins, which involves recruiting inflammatory cells to restore tissue homeostasis. In the second phase, re-epithelization begins, with the proliferation and migration of keratinocytes in the epidermis. While in the dermis, the granulation tissue is formed by fibroblasts depositing an extracellular matrix. Subsequently, the remodeling phase initiates, with the deposition of a permanent extracellular matrix, mainly collagen, which can last up to 1 year depending on the size of the wound and extension.^6^

In addition, mast cells are immune cells in the skin that play a role in maintaining immunity, but when there is an injury to the skin, they stimulate the influx of leukocytes by releasing inflammatory responses.^7^ These cells have heparin and histamine in their granules, the latter being a trigger for allergic processes.^8^ In the repair of injuries, inflammation is an important process that prevents infections; however, the reduction of inflammatory events has been shown to improve wound repair. In experimental models in which there was a decrease in inflammation, a smaller, functionally better scar was obtained,^9,10^ suggesting that modulation of the local immune response may be a therapeutic strategy.^11,12^

Coumarin, also known as benzopyran-2-one or chromen-2-one, is a class of organic compounds characterized by the fusion of aromatic rings with 1,2-pyran. This class has a variety of compounds, which are present in various natural sources, but can also be easily synthesized, resulting in new and diverse coumarin derivatives.^13^ Coumarin and its derivatives have shown significant pharmacotherapeutic potential in several biological activities, such as antioxidants,^14,15^ antidepressants,^16,17^ anti-HIV,^18,19^ anticancer,^20,21^ anti-inflammatory,^22,23^ and anticonvulsant.^24,25^ Pharmacological activities, as well as the therapeutic applications of coumarins, can change depending on the pattern of substitution in different positions of the ring.^26^

The anti-inflammatory activity of coumarins in repairing skin wounds has not yet been demonstrated. However, it has been shown to reduce edema in rabbit ears^27^ and mouse paws.^28,29^ Therefore, the insolubility of some compounds in water restricts the application of drugs in some biological studies. In addition, poorly soluble drugs have bioavailability problems, with dissolution being the limiting factor for their absorption in the body. Considering that coumarins, in general, are practically insoluble in water, the formation of a drug/cyclodextrin (CD) inclusion complex can enhance their pharmacological properties, increasing their effectiveness and safety. Moreover, the supramolecular interaction between the species can increase the aqueous solubility of the drug, increase the chemical and physical stability, and improve the delivery of the drug through biological systems.^30^

In this study, we aimed to develop two different synthetic coumarins and β-CD inclusion complexes for evaluation of the activity of these structured compositions in the tissue repair and acceleration of the healing process of skin wounds in mice. The coumarin/β-CD complexes were studied using experimental and theoretical methods to provide a deeper foundation for determining the feasibility of developing novel coumarin inclusion complexes for repairing skin wounds.

## 2 Experimental Section

### 2.1 Reagents and Solvents

Dimethyl sulfoxide (DMSO), β-CD, sulfuric acid, *m*-cresol, 3-methoxyphenol, and ethyl acetoacetate were acquired from Sigma-Aldrich (St. Louis, MO). Ethanol HPLC grade was purchased from J.T. Baker (Mexico City, DF, Mexico), and purified water was obtained using the Millipore Milli-Q Plus system (Bedford, MA).

The coumarins 4,7-dimethyl-2*H*-chromen-2-one (DMC) and 7-methoxy-4-methyl-2*H*-chromen-2-one (MMC) were synthesized by the Pechmann method.^31^ Briefly, a mixture of ethyl acetoacetate (1.5 mmol) and sulfuric acid 80% (1.0 mL) was stirred at room temperature (25 ± 3 °C) for 30 min, and further, 1.0 mmol of the corresponding phenol (*m*-cresol for DMC and 3-methoxyphenol for MMC) was added and the solution was kept at room temperature (25 ± 3 °C) for 18 h. After cooling, the mixture was poured over crushed ice, and the solid formed was filtered and dried. DMC and MMC were obtained with excellent isolated yields (quantitative and 80%, respectively). The spectral data (^1^H NMR and ^13^C NMR) (Figure S1) of coumarins are as follows: (i) DMC: ^1^H NMR (400 MHz, CDCl_3_): δ 7.46 (d, *J* = 8.0 Hz, 1H, H-5); 7.12 (s, 1H, H-8); 7.09 (d, *J* = 8.0 Hz, H-6); 6.25 (s, 1H, H-3); 2.43 (s, 3H, CH_3_), 2.40 (s, 3H, CH_3_) ppm; ^13^C NMR (100 MHz, CDCl_3_): δ 161.1 (C-2), 153.6 (C-8a), 152.4 (C-4a), 142.8 (C-4), 125.3 (C-6), 124.2 (C-5), 117.6 (C-8 or C-7), 117.2 (C-8 or C-7), 114.0 (C-3), 21.6 (CH_3_), 18.6 (CH_3_) ppm; (ii) MMC: ^1^H NMR (400 MHz, CDCl_3_): δ 7.48 (d, *J* = 8.8 Hz, 1H, H-5), 6.83 (dd, *J* = 8.8 and 2.4 Hz, 1H, H-6), 6.80 (d, *J* = 2.4 Hz, 1H, H-8), 6.12 (s, 1H, H-3), 3.87 (s, 3H, OCH_3_), 2.38 (s, 3H, CH_3_) ppm; ^13^C NMR (100 MHz, CDCl_3_): δ 162.7 (C-7), 161.3 (C-2), 155.3 (C-8a), 152.6 (C-4a), 125.5 (C-5), 113.6 (C-4), 112.3 (C-6), 112.0 (C-3), 100.9 (C-8), 55.8 (OCH_3_), 18.9 (CH_3_) ppm.

### 2.2 Preparation of Inclusion Complexes

The inclusion complexes were prepared in molar ratios of 1:1 and 1:2 of β-CD/coumarins in two ways. First, MMC or DMC solutions were prepared using ethanol due to their better solubility, and β-CD was dissolved in water. These solutions were left for 1 min in an ultrasonic bath for complete solubilization, and then, the β-CD solution was poured into the guest molecule solution, which was left under agitation at 600 rpm for 24 h. The final solutions were taken to the rotary evaporator for evaporation of the organic solvent, the residue was dissolved in ultrapure water, and after filtration, they were frozen and lyophilized (FL).^32^ These complexes in molar ratios of 1:1 and 1:2 of β-CD/coumarins were named DMC@β-CD (FL), (DMC)_2_@β-CD (FL), MMC@β-CD (FL), and (MMC)_2_@β-CD (FL). Second, for comparison, mechanical mixtures (MMs) were also synthesized in 1:1 and 1:2 molar ratios of β-CD/coumarins, respectively. The β-CD and the guest molecules (DMC and MMC) were weighed, homogenized with the aid of a pestle, and placed for subsequent characterizations. These systems were named DMC@β-CD (MM), (DMC)_2_@β-CD (MM), MMC@β-CD (MM), and (MMC)_2_@β-CD (MM).

### 2.3 Determination of Inclusion Yield (IY) and Inclusion Ratio (IR)

The IY and IR for inclusion complexes with DMC and MMC were calculated by using the following formulas
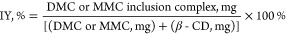

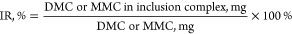


The IY may be immediately acquired from the preparation process of inclusion complexes. To obtain the IR, the inclusion complex (5 mg) from the product was dissolved in ultrapure water (20 mL), and after determination by HPLC-DAD,^33^ the amounts of DMC and MMC in 5 mg of complexes were recorded. Based on the amount of the product, the IR was obtained by using the above-mentioned formula.

### 2.4 Apparatus and Conditions

The physical-chemical characterization allows the investigation and evaluation of the molecular interaction between the coumarins and β-CD molecules, aiming to evaluate the alterations of the physicochemical properties of the complexes based on the noncomplexed molecules. Fourier transform infrared (FTIR) spectroscopy was performed using a PerkinElmer Spectrum 400 spectrometer, operating between 4000 and 600 cm^–1^, with a resolution of 4 cm^–1^, in which the samples were prepared using the KBr tablet method. Thermogravimetric analysis (TGA) and differential thermal analysis (DTA) were performed using a Shimadzu DTG-60H thermobalance with a heating rate of 10 °C min^–1^ between 25 and 700 °C under a nitrogen flow (50 mL min^–1^), and the samples were packed in an alumina crucible in the powder form. Isothermal titration calorimetry (ITC) analyses were performed in a microcalorimeter VP-ITC (Microcal, Malvern) to determine the physicochemical parameters of interactions between β-CD and DMC or β-CD and MMC. Experimental concentrations used in all titrations between the guest (DMC or MMC in the syringe) and host (β-CD into the sample cell) were 20.0 and 1.0 mmol^–1^, respectively. Titrations were performed with 25 injection points each, at an interval of 350 s, with 220 rpm stirring, and at the temperature of 298.15 K. The first injection of each titration was of 1 μL to remove the effects of dispersion, and the successive ones were of 10 μL each. Titrations were also carried out between each titrant and DMSO, or DMSO with each titrant, to remove the effects of the interaction between the compounds and the solvent. Graphics obtained were analyzed by software Origin 9, which uses the least-squares nonlinear regression model in the curve fitting, applying the Wiseman isotherm.^34^ Thereby, all physicochemical parameters can be obtained by standard equations, and these are presented as the means and standard deviation of at least three titration processes. The solid-state 13C nuclear magnetic resonance (NMR) spectra were recorded at room temperature in a Varian-Agilent 400 MHz spectrometer operating at 100.52 MHz (corresponding to a magnetic field of 9.4 T). A triple-resonance radiofrequency (RF) probe head with 4 mm diameter zirconia rotors was used for experiments with cross-polarization (CP), 1H decoupling (SPINAL pulse sequence), and magic angle spinning (MAS) at the spinning frequency of 10 kHz. In the CP pulse sequence, the 1H π/2 pulse duration was 3.6 μs, the spectral window was 50 kHz, the recycle delay was 5 s, and the number of accumulated transients was typically around 2000. Chemical shifts, given in parts per million (ppm), were externally referenced to tetramethylsilane (TMS), using the methyl peak (at 17.3 ppm) in the 13C NMR spectrum of hexamethylbenzene (HMB).

### 2.5 Computational Details for CD Inclusion Complexes

Density functional theory (DFT) calculations were performed to obtain topological parameters and energetic properties of inclusion complexes formed from the inclusion process of two coumarin analogues (guests) into the β-CD cavity (host). All calculations were carried out in a DMSO medium, taking into account two distinct stoichiometric arrangements: 1:1 (one β-CD for one guest molecule): DMC@β-CD and MMC@β-CD; 1:2 (one β-CD for two guest molecules): (DMC)_2_@β-CD and (MMC)_2_@β-CD; and 2:1 (two β-CDs for one guest molecule): DMC@(β-CD)_2_ and MMC@(β-CD)_2_ (Figure S2).

The initially guessed geometries for all isolated guests (DMC and MMC) and β-CD were fully optimized with the B97D^35^ functional, using the Pople’s standard split valence 6-31G(d,p) basis set.^36^ The B97D functional, employed in conjunction with Pople's ubiquitous 6-31G(d,p) split-valence basis set, was utilized to conduct a comprehensive geometry optimization of the initial guess structures for all isolated guest molecules (DMC and MMC) as well as the β-CD host. Notably, the B97D functional inherently accounts for dispersion interactions through its formulation, which is a critical factor for accurate characterization of supramolecular systems.^35^ B97D/6-31G(d,p) harmonic frequencies were calculated for the host and guests in their isolated forms, identifying them as true minima (all frequencies were real) on the potential energy surface (PES). Afterward, six distinct inclusion complex arrangements in 1:1, 1:2, and 2:1 molar ratios were designed considering the two coumarins and β-CD. All of the arrangements had their geometries fully optimized at the B97D/6-31G(d,p) level of theory, and from the harmonic frequency analysis, all six complex geometries were also characterized as true minima on the PES.

Within the quantum mechanical formalism, the solvent effect was considered using the solvation model based on density (SMD).^37^ In the condensed phase, the presence of the solvent is replaced by its dielectric constant (for DMSO ε = 46.8). The solute was placed in a cavity of suitable shape to enclose the whole molecule, which was immediately considered in the continuum dielectric. All theoretical calculations were carried out using the Gaussian 09 quantum mechanical package.^38^

### 2.6 *In Vivo* Wound Healing Assay

#### 2.6.1 Mice Studies and Ethical Considerations

All mice studies were conducted under specific pathogen-free conditions. Swiss mice (male, 8 weeks old) were maintained in the animal breeding unit at the Departamento de Ciências Naturais, Universidade Federal de São João del-Rei (UFSJ), Brazil. The animal care and handling procedures were in accordance with the guidelines of the Institutional Animal Care and Use Committee, and the study received prior approval from the Ethics Committee in Animal Experimentation (CEUA/UFSJ protocol number: 051/2017). Each group contained six mice per time point and was randomly grouped.

#### 2.6.2 Wounding

Mice were anesthetized with ketamine (97 mg kg^–1^) and xylazine (15 mg kg^–1^), and their dorsal thoracic skin was shaved and cleaned with 70% ethanol before wounding. Two circular through-and-through full-thickness excisional wounds (each with 7.0 mm diameter, 0.49 cm^2^ area) were made by picking up a fold of skin and using a biopsy punch, resulting in the generation of one wound on each side of the midline. Mice were caged individually, and lesions were left unsutured to allow the evaluation of the process of healing by secondary intention.^10^ Male mice between 8 and 10 weeks of age of the Swiss lineage received the following: (i) 20 μL of saline solution, or (ii) 20 μL of (DMC)_2_@β-CD (FL) at 1 mg mL^–1^, or (iii) (MMC)_2_@β-CD (FL) solution at 1 mg mL^–1^ for five consecutive days in an interval of 24 h.

#### 2.6.3 Histology

Mice were euthanized by lethal doses of anesthetics at 7 or 60 days, shaved when necessary, and the skin with the lesions was dissected. One of the sores was fixed in Carson’s altered Millonig’s phosphate-buffered formalin for 24 h, oppositely segmented in half and the different pieces were dried out in ethanol and implanted in paraffin for histological examinations, keeping the guideline conventions. Serial 5 μm transverse sections from the middle of the wound were stained with hematoxylin and eosin (H&E), alcian blue safranine, or Gomori′s trichrome. Each group contained six mice per time point, and we analyzed one section per mouse, per time point, resulting in six sections per time point.

#### 2.6.4 Morphometry

The histological images of the slides were captured by a digital camera (Moticam 3000) coupled to an optical microscope (Olympus BX51), obtaining images at 40, 100, and 400× magnifications. Mast cells were identified with alcian blue safranine staining to evaluate the maturation. Mast cells were counted in 10 fields of 10,000 μm^2^ within the wound healing area of one section per mouse, and the results from six sections per group were expressed as the mean ± standard error of the mean (SEM). Within the wound healing area, results from one section per mouse and from six sections per group were expressed as the mean ± SEM. The mature granules of mast cells express a greater number of proteases, showing a red color, and the immature granules express proteoglycans, showing a blue color and those with intermediate maturation have a purple color.^8^

#### 2.6.5 Macroscopic Analysis

Wounds or the healed area were photographed with an in-picture ruler for scale using a digital camera (Sony DSC-F717, Tokyo, Japan) immediately after and at days 60 post wounding. Wound outlines were manually traced for calculation of wound area and scar area using image analysis software ImageJ. Each group contained six mice per time point, and each mouse had two wounds, resulting in 12 measures per time point.

### 2.7 Binging Affinities of DMC and MMC with Target Proteins

The chemical structures of DMC and MMC, previously optimized and with the partial charges of the atoms determined at the same level (Section 2.5), were used in molecular docking studies, with the proteins tumor necrosis factor (TNF-2, PDB code: 2AZ5); arachidonate 5-lipoxygenase (ALOX5, PDB code: 6N2W); cyclooxygenase (COX-1, PDB code: 6Y3C; COX-2, PDB code: 5IKR); mitogen-activated protein kinase (MAPK1, PDB code: 4QTA; MAPK3, PDB code: 4QTB),^39^ taking into account the same procedures employed previously.^40,41^ According to our calculation protocol, a radius of about 10 Å was considered, where the residues were kept as flexible. Due to the nature of the docking methods, the calculations were carried out, generating 50 poses (conformation and orientation) for each ligand investigated.

### 2.8 Software and Statistical Methods

Software ChemDraw (PerkinElmer Informatics) was used to draw the chemical structures, OriginPro 9.0 (OriginLab) was used to perform the mathematical calculations and graphical plotting of the data, MestReNova (MestreLab Research S. L) was used to analyze the NMR spectra, and the software package Molegro Virtual Docker program (MVD) was for molecular modeling. The statistical significance of differences between groups of morphometry was determined using one-way analysis of variance (ANOVA), followed by the Student–Newman–Keuls test, using GraphPad Prism 7 (GraphPad Software, San Diego, CA). Values of *p* ≤ 0.05 were considered significant. The results are expressed as mean ± SEM.

## 3 Results and Discussion

### 3.1 Solubilization *versus* Skin Drug Permeation

The stratum corneum is considered the principal barrier for the permeation of drug across the skin. Transdermal delivery is limited to drugs with a favorable partition coefficient and low molecular weight. Despite the low molecular weight, DMC and MMC have poor solubility and high lipophilicity, as indicated by their log* P* values (log *P*_DMC_ = 1.806 and log *P*_MMC_ = 1.890).^42^ In order for a drug to cross the stratum corneum layer, it needs to be in the solubilized state, which can be achieved with inclusion complexes of the drug with CDs. This solubilization can enhance drug permeation, improving the aqueous drug solubility required for penetration across viable skin tissues that are rich in water. DMC and MMC inclusion complexes with β-CD can amplify the thermodynamic driving force for transdermal permeation. Additionally, the protein structure of the stratum corneum can be disrupted by high concentrations of CDs, facilitating transdermal penetration of the solubilized compounds. However, CDs can both enhance and hamper drug permeation through biological membranes; therefore, it is important to understand the entire complexation process.^43,44^

### 3.2 ITC, IY, and IR

**Figure 1:**
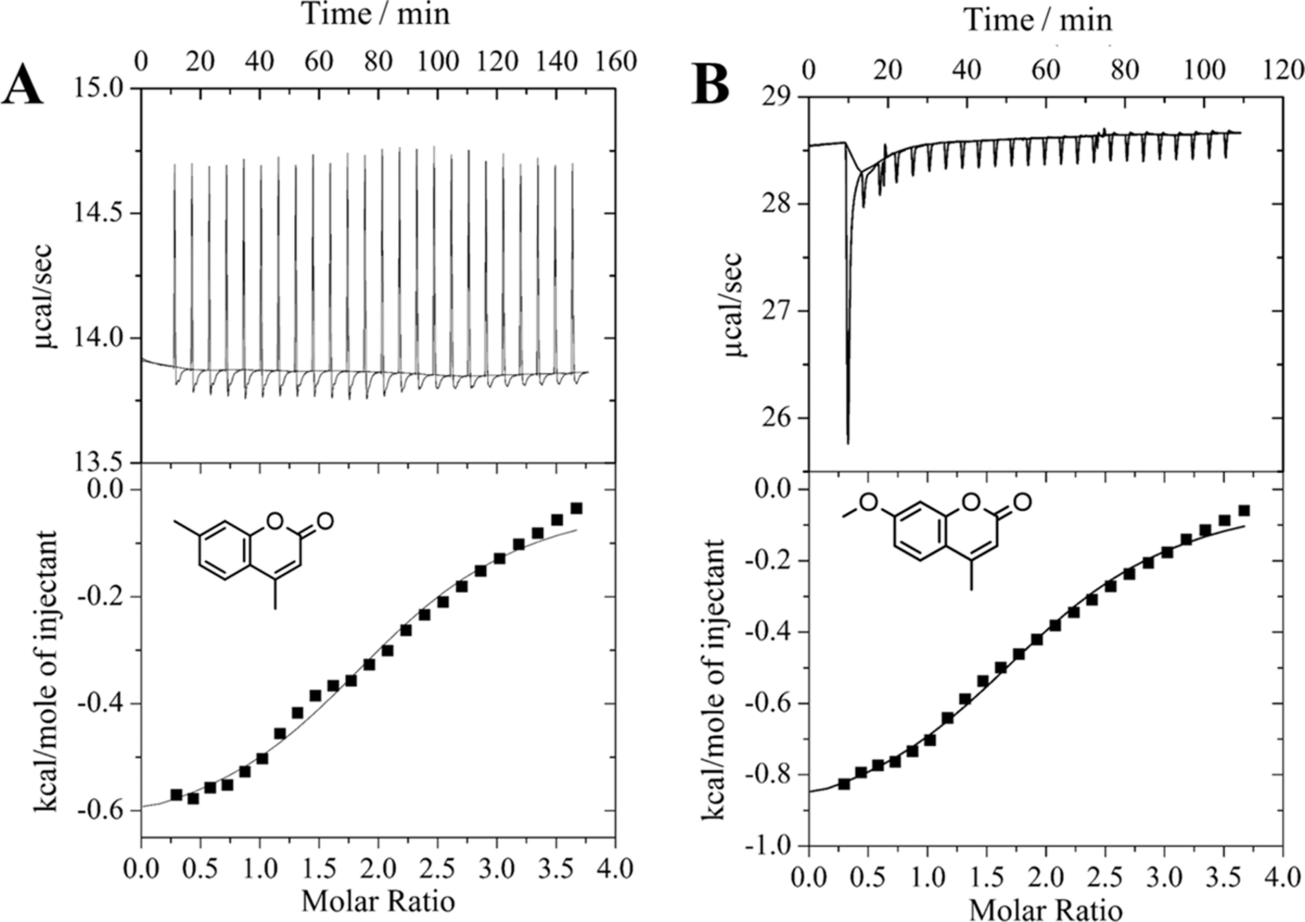
ITC for (A) DMC and (B) MMC titration with β-CD.

Understanding the intriguing equilibrium involved in the CD molecular recognition processes toward guest molecules requires knowledge about the structural and energetic features associated with these events. Thus, ITC has been demonstrated to be a valuable thermodynamic approach to investigating host–guest interactions based on CDs, especially when it is associated with structural analysis (by experimental and/or theoretical calculations).^45,46^Figure 1 shows the titration curves for DMC in β-CD and MMC in β-CD systems after the subtraction of the dilution curve. Thermodynamic parameters obtained by DMC or MMC titration into the β-CD solution are presented in Table 1.

**Table 1:** Thermodynamic Parameters for the DMC or MMC (at 20.0 mmol L^–1^) Interaction with β-CD (at 1.0 mmol L^–1^) at 298.15 K in DMSO

supramolecular system	*n*	*K*	Δ*H*° (kJ mol^–1^)	*T*Δ*S*° (kJ mol^–1^)	Δ*G*° (kJ mol^–1^)
DMC@β-CD	2.11 ± 0.09	3606.7 ± 166.5	–2.5 ± 0.4	17.9 ± 0.2	–20.4 ± 0.1
MMC@β-CD	2.02 ± 0.04	3565.0 ± 91.9	–4.2 ± 0.2	16.1 ± 0.1	–20.3 ± 0.1

Based on these results, the stoichiometry for both supramolecular systems is related to the interaction of two guest molecules (DMC or MMC) with one β-CD cavity, forming a 1:2 host/guest system, *i.e.*, (DMC)_2_@β-CD and (MMC)_2_@β-CD. The absence of difference between the molar ratio of these supramolecular systems can be associated with the similar chemical structure of DMC and MMC guest molecules. In this sense, similar binding constants (*K*) were also observed for both systems, which could be correlated to guest volume and host cavity size.^47^ These *K* values are in a similar magnitude as other CD interactions with small guest molecules.^48^

Although both supramolecular systems were spontaneously formed, since the standard Gibbs free energy (Δ*G*°) is similar for DCM and MMC supramolecular systems, the entropic and enthalpic contributions for these systems are different. For the DMC system, the entropic variation (*T*Δ*S*°) is greater than that observed for the MMC and β-CD one; however, in both cases, positive values can be related to the 1:2 host/guest, supramolecular structure. Usually, the large positive *T*Δ*S*° can be ascribed to the significantly important translational and conformational freedoms of the host and guest upon complexation. In these DMC and MMC situations, these phenomena could be related to the reduced interaction between guest molecules (DMC or MMC) in solution. Moreover, these processes in which *T*Δ*S* is positive can be related to the hydrophobic interaction between the host and the guest.^49^

The higher enthalpic contribution for the MMC could be associated with the substituted group in the aromatic ring, which is able to increase the electronic density in the ring, favoring van der Waals interactions between the host cavity and the guest molecule. Similar results have been reported in which several substituted groups were evaluated.^50,51^ Additionally, the MMC guest is able to form a hydrogen bond with β-CD hydroxyl groups, which were confirmed by theoretical calculations described below.

In addition, using the ratio of β-CD and DMC or MMC of 1:2, a stirring speed of 600 rpm, a reaction temperature of 25 ± 3 °C, and a reaction time of 24 h, IYs of 95.8 and 93.6% for DMC and MMC, respectively, were obtained. The inclusion ratios for DMC and MMC were 94.6 and 92.5%, respectively.

### 3.3 Theoretical Approach for Inclusion Complexes

The main goal of the theoretical calculation work was to provide the topology and energetic parameters of the inclusion complexes formed from DMC and MMC as well as β-CD, which can be used to predict the most favorable complex arrangement. A detailed understanding of the interactions between the guests and hosts at the molecular level can be very useful to support the interpretation of experimental findings.

**Figure 2:**
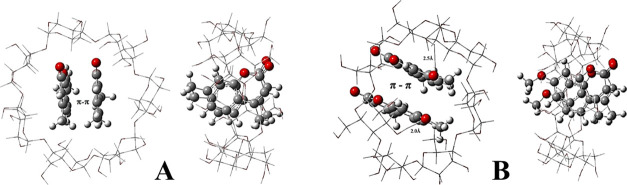
Inclusion complex geometries optimized at the B97*D*/6-31G(d,p) level of theory: (A) (DMC)_2_@β-CD and (B) (MMC)_2_@β-CD.

The data of B97D energetic properties, *i.e.*, electronic complexation energy (Δ*E*) and Gibbs free energy (Δ*G*), calculated in the DMSO medium for the inclusion complexes formed between the DMC and MMC with β-CD in molar ratios of 1:1, 1:2, and 2:1, are given in Table 2. Among the three arrangements studied (1:1, 1:2, and 2:1), the global minimum found in the equilibrium is the 2:1 (MMC)_2_@β-CD inclusion complex because it is the most energetically favored orientation (see Table 2). For the 2:1 investigated complexes, the higher stability found for the (MMC)_2_@β-CD complex is related to the formation of two hydrogen bonds established between the oxygen of the MMC methoxy group and the secondary hydroxyls of the β-CD, as can be seen in Figure 2. These two hydrogen bonds, along with π–π stacking interactions, can be considered the driving forces responsible for stabilizing this inclusion complex arrangement. All of the other B97D optimized structures of inclusion complex arrangements are depicted in Figure S3.

**Table 2:** B97*D*/6-31G(d,p) Electronic Complexation Energy (Δ*E*) and Gibbs Free Energy (Δ*G*) Calculated in the DMSO Medium, for the Inclusion Complexes Formed from DMC, MMC, and β-CD

stoichiometry	inclusion complexes	Δ*E*_DMSO_ (kcal mol^–1^)	Δ*G*_DMSO_ (kcal mol^–1^)
1:1	DMC@β-CD	–2.7	13.7
	MMC@β-CD	–5.2	11.1
1:2	(DMC)_2_@β-CD	–14.6	–4.9
	(MMC)_2_@β-CD	–18.4	–5.8
2:1	DMC@(β-CD)_2_	–7.8	–1.1
	MMC@(β-CD)_2_	–9.5	–1.8

With these theoretical findings, structural and energetic parameters can be described involving the inclusion complex of coumarin analogues and β-CD at a molecular level, which were in good agreement with the experimental data. The theoretical results obtained in the DMSO medium corroborate with ITC data regarding the stoichiometry of the complexes (1:2, host/guest), the small difference between the Δ*G* values of the complexes, as well as whether the MMC complexes are somewhat more stable than those with DMC.

### 3.4 FTIR

FTIR technique is an important tool in the characterization of inclusion complexes as it assesses the variation in the dipole moment of chemical bonds due to the vibrations of atoms. Thus, new intermolecular interactions can be evaluated due to the penetration of the functional groups of the guest molecule into the CD cavity.^52,53^ FTIR can be used to compare the spectra of the pristine drug and β-CD, besides FL and MM, to evaluate the displacements, overlaps, and an increase or reduction of the intensity of the bands due to interactions between groups of atoms of the molecules under study.

**Figure 3:**
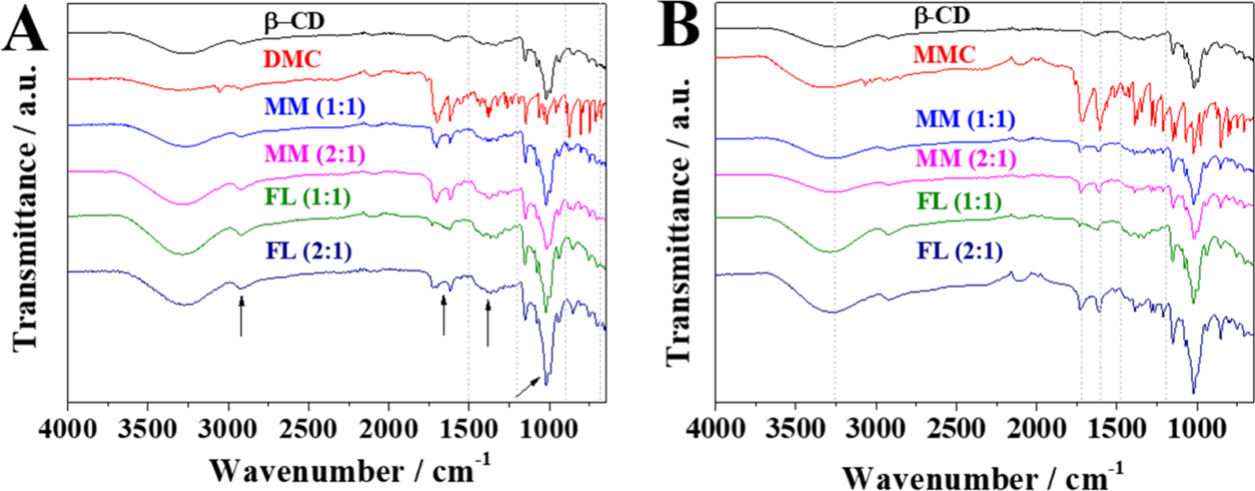
Infrared spectra at 4000–600 cm^–1^ of (A) DMC, β-CD, MM (1:1), MM (2:1), FL (1:1), and FL (2:1); and (B) MMC, β-CD, MM (1:1), MM (2:1), FL (1:1), and FL (2:1).

Figure 3A shows the infrared spectra of MMs and FLs in the proportions 1:1 and 2:1, besides β-CD and DMC, in the range from 4000 to 600 cm^–1^. Table 3 shows the main bands observed by FTIR spectroscopy of β-CD, DMC, and their systems (MMs and FLs). It can be seen that there is no significant change in MMs 1:1 and 2:1 and also in FLs 1:1 and 2:1, and all present overlapping bands of β-CD and DMC. In addition, the profile of MMs and FLs in the region from 1500 to 1190 cm^–1^ is very different from that of the separated materials, both in the presence of the bands and in their intensity. Analyzing the spectra of the inclusion complexes, it can be seen that there are some changes when compared with the spectra of the individual materials (marked with arrows in Figure 3A), notably the intensification of the band in 2920 cm^–1^ referring to the C–H stretch, and the attenuation and displacement of the bands in 1730, 1619, and 1551 cm^–1^ referring to the asymmetric stretching of the C=O bond and the stretching of the C=C bond of alkenes. In addition, there is a change in the profile of the bands in the region from 947 to 697 cm^–1^ associated with β-CD C–H bonds. The observed changes show a great reduction or disappearance of the characteristic bands of DMC, indicative of strong interactions between the guest molecule and the β-CD.^30,54^

**Table 3:** Main Bands Observed by FTIR of β-CD, DMC, MM (1:1), MM (2:1), FL (1:1), and FL (2:1)

	wavenumber (cm^–1^)					
tasks	β-CD	DMC	MM (1:1)	MM (2:1)	FL (1:1)	FL (2:1)
ν(O–H)	3265	3307	3277	3290	3290	3270
ν(C–H)	2920	2917	2916	2922	2927	2922
δO–H, water of hydration	1640				1643	
δ(C=O) ketone		1700	1700	1700	1735	1727
υ(C=C) aromatic ring		1617, 1552	1619, 1554	1617, 1556		
δ_ass_(C–H), δ(C–OH)		1379	1453, 1375		1453, 1375	
δ(C–O–H), ν(C–O–C), ν_ass_(C–O–C)	1380, 1154, 1019	1327, 1154, 1017	1327,1154, 1022	1327, 1154, 1017	1327, 1154, 1025	1332, 1151, 1019
δ(C–H)	853, 752		854, 752	857, 751	854, 753	851, 754

According to the spectra in Figure 3B and the data in Table 4, in general, there were also no significant changes in the positions of the main bands of the host and guest molecules, and there was an overlap of the bands of the separated materials. Analyzing the spectrum of (MMC)_2_@β-CD (FL), it can be seen that it has a different profile from the other spectra according to the bands highlighted with a dashed line, that is, there is a thinning of the β-CD band attributed to the O–H vibration at 3265 cm^–1^ due to the formation of a new hydrogen-bond pattern. Modification in the intensity and displacement of the bands at 1729 and 1608 cm^–1^ and a change in profile, positions, and intensities of the bands between 1394 and 1207 cm^–1^ were observed when compared with the spectra β-CD and MMC. These changes suggest the formation of the inclusion complexes 1:1 and 2:1, with the 2:1 complex presenting a band related to the connections in a different way from other MMs and FLs with 1:1.^30^

**Table 4:** Main Bands Observed by FTIR of β-CD, MMC, MM (1:1), MM (2:1), FL (1:1), and FL (2:1)

	wavenumber (cm^–1^)					
tasks	β-CD	MMC	MM (1:1)	MM (2:1)	FL (1:1)	FL (2:1)
ν(O–H)	3265	3313	3289	3265	3289	3283
ν(C–H)	2920		2917	2929	2922	2918
δ(C=O) ketone		1712	1729	1722	1733	1729
δO–H, water of hydration	1638					
υ(C=C)		1604, 1507	1610	1613	1619	1608
δ_ass_(C–H), δ(C–OH)		1390		1390		1390
ν(C–O–C), ν_ass_(C–O–C)	1149, 1021	1153, 1023	1154, 1021	1151, 1023	1151, 1021	1151, 1023
δ(C–H)	846	851	853	855	853	855

### 3.5 TGA and DTA

TGA assesses the change in the mass of the compound as a function of temperature, enabling the observation of processes such as decomposition and dehydration, phenomena that are indicative of the stability of substances.^55^ DTA is a technique in which the temperature difference between the samples and a reference material (thermally inert) is measured as a function of temperature, and for that, a differential thermal curve is recorded, which allows one to determine the nature of events (endothermic/exothermic). These two techniques are used for the characterization of inclusion complexes since the formation of the complex leads to changes in the thermal behavior, which are compared with the precursors.^56^

**Figure 4:**
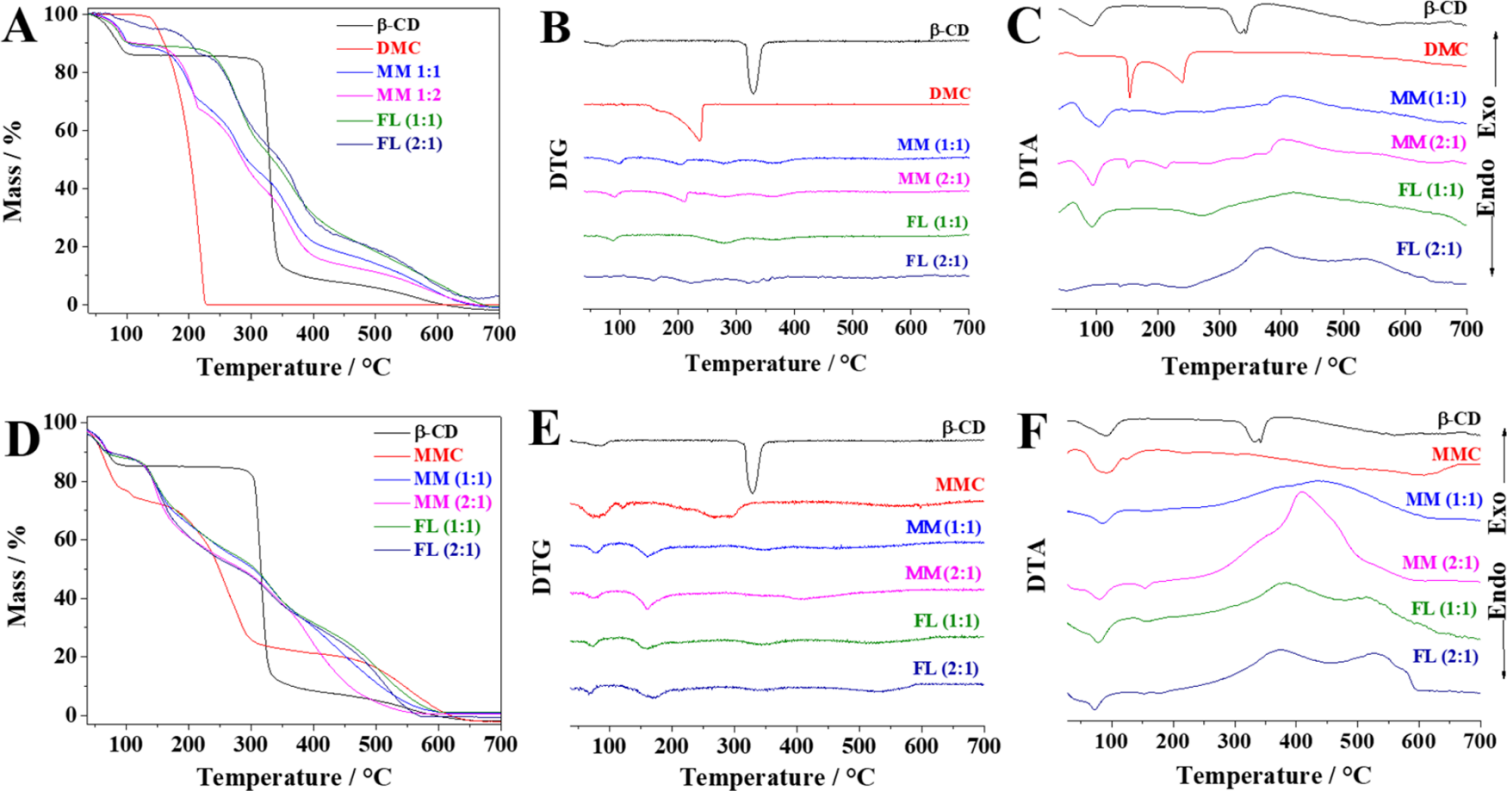
Curves of (A and D) TG, (B and E) DTG, and (C and F) DTA of DMC or MMC and their inclusion complexes by MMs and FL in guest/host ratios of 1:1 and 2:1.

TG curves of β-CD, DMC, and inclusion complexes by MMs and FLs are shown in Figure 4A–C, and the events with temperatures and percentages of mass losses of substances are shown in Table 5. In Figure 4A,B, three events can be observed: the first is in the range of 58–108 °C, referring to the loss of water molecules (10% of mass); the second is a narrower and more intense event in the range of 305–355 °C representing a loss of 75% of mass related to the decomposition of β-CD; and the last event represents successive events that occur in the range of 356–646 °C related to decomposition with complete calcination of the compost.^57,58^ In the DTA curve (Figure 4D), three events were observed: the first is an endothermic peak with the *T*_max_ at 91 °C, attributed to dehydration of β-CD; the second endothermic peak has two *T*_max_, 330 and 342 °C, due to the decomposition steps taking place at different speeds; and in the final one, an endothermic event is perceived, which represents the last steps of decomposition and calcination.^57^

**Table 5:** Main Thermal Events Attributed to β-CD, DMC, MM (1:1), MM (2:1), FL (1:1), and FL (2:1)

	TGA and DTG					DTA			
	events					events			
compound	*T*_initial_ (°C)	*T*_final_ (°C)	*T*_max_ (°C)	Δ*m* (%)	tasks	*T*_initial_ (°C)	*T*_final_ (°C)	*T*_max_ (°C)	tasks
β-CD	58	108	83	15	dehydration	38	119	91	endothermic dehydration process
	303	355	330	75	decomposition	299	360	330, 342	endothermic fusion and decomposition processes in successive stages of β-CD
	356	646	501	10		394	700	571	
DMC	146	247	237	100	decomposition	134	257	153, 240	endothermic decomposition processes in successive stages
MM (1:1)	53	109	99	10	dehydration	59	137	102	endothermic dehydration process
	157	216	201	20	decomposition	166	237	207	endothermic decomposition processes of DMC
	251	309	280	25		244	313	281	
	327	402	360	25		382	455	410	exothermic process of breakdown of interactions between DMC and β-CD
	469	694	531	20		456	700		endothermic decomposition process of MMs
MM (2:1)	59	106	90	10	dehydration	57	127	92	endothermic dehydration process
	147	219	211	24	decomposition	145	168	153	endothermic decomposition process of DMC
	219	316	281	26		175	225	212	
	321	414	363	25		330	375	360	exothermic process of breakdown of interactions between DMC and β-CD
	416	662	604	15		380	603	403	
FL (1:1)	60	106	88	10	dehydration	61	135	90	endothermic dehydration process
	208	318	280	34	decomposition	215	325	270	endothermic decomposition process of DMC
	326	433	366	32		337	541	420	exothermic process of breakdown of interactions between DMC and β-CD
	441	679	630	24		561	700		endothermic decomposition process of the inclusion complex
FL (2:1)	64	93	77	3	dehydration	30	86	56	endothermic dehydration process
	156	223	207	12	decomposition	193	276	240	endothermic decomposition processes of DMC
	224	315	272	28		306	459	373	exothermic process of breakdown of interactions between DMC and β-CD
	323	437	372	33		473	639	537	
	450	664	582	24					

Analyzing the DMC thermogravimetric curves, a single 100% mass loss event between 146 and 247 °C is perceived, attributed to the complete decomposition of the compound, and through the DTA curve (Figure 4D), it is observed that this event occurs in two endothermic decomposition processes with *T*_max_ at 153 and 240 °C. TG and DTG curves of DMC@β-CD (MM) and (DMC)_2_@β-CD (MM) were very similar, with subtle differences in the loss of mass and temperature. In general, both present 10% loss of mass due to dehydration and then successive decomposition events that may be related to the disruption of β-CD and DMC bonds and then β-CD decomposition since the guest molecule degrades at a lower temperature. In the DTA curve, however, they have a small difference in endothermic and exothermic processes since DMC@β-CD (MM) in its last decomposition process occurs endothermically, and (DMC)_2_@β-CD (MM) occurs exothermically. The difference in the thermal curves of the MMs when compared to those of the separate compounds can be attributed to the formation of intermolecular bonds during maceration. TG curves of DMC@β-CD (FL) and (DMC)_2_@β-CD (FL) have a very similar profile from 140 °C onward, and the beginning of the two curves presents different events of dehydration and decomposition, inferring a greater amount of DMC for (DMC)_2_@β-CD, which starts to degrade at lower temperatures, marked by successive events (Figure 4B). When compared with the separate compounds (β-CD and DMC), it was noticed that MM and FL have a totally different profile, indicating that weak interactions between the two compounds occurred.

TG curves of β-CD, MMC, and inclusion complexes by MMs and FLs are shown in Figure 4D–F, and the events with temperatures and percentages of mass losses of the substances are shown in Table 6. In the TG/DTG curves of MMC, four endothermic events were observed (Figure 4D,E). The first event occurred at 110 °C due to loss of water molecules; the second event involved a very small loss of 5% of mass; the third event, which involved a two-stage endothermic decomposition with a loss of 46.5% of mass, occurred at a temperature between 174 and 329 °C; and the last event occurred slightly up to 700 °C, which led to the final decomposition of the coumarin compost. MMC@β-CD (MM) and (MMC)_2_@β-CD (MM) have similar thermal profiles until the temperature of 161 °C, in which two events were observed: one involved dehydration of the compounds at *T*_max_ equal to 78 °C and the second event represented the beginning of decomposition of DMC present in physical mixtures (*T*_max_ = 161 °C). However, upon comparison of the thermal curves of TG/DTG (Figure 4D,E) and DTA (Figure 4F), it was noticed that there are some differences after 161 °C, mainly because (MMC)_2_@β-CD (MM) starts to decompose at slightly lower temperatures than MMC@β-CD (MM). In addition, (MMC)_2_@β-CD (MM) presents a broad and more acute exothermic peak (*T*_max_ = 408 °C) than MMC@β-CD (MM), which presents a broad peak in *T*_max_ equal to 439 °C, followed by a final endothermic decomposition.

**Table 6:** Main Thermal Events Attributed to β-CD, MMC, MM (1:1), MM (2:1), FL (1:1), and FL (2:1)

	TGA and DTG					DTA			
	events					events			
compound	*T*_initial_ (°C)	*T*_final_ (°C)	*T*_max_ (°C)	Δ*m* (%)	tasks	*T*_initial_ (°C)	*T*_final_ (°C)	*T*_max_ (°C)	tasks
β-CD	58	108	83	15.0	dehydration	38	119	91	endothermic dehydration process
	303	355	330	75.0	decomposition	299	360	330, 342	endothermic fusion and decomposition processes in successive stages of β-CD
	345	646	501	10.0		394	700	571	
MMC	32	110	78	25.0	dehydration	43	153	91	endothermic dehydration processes
	111	130	121	5.0	decomposition				
	174	329	268	46.5		322	666	606	endothermic decomposition processes in successive stages
			298						
	465	665	584	23.5					
MM (1:1)	52	98	78	12.5	dehydration	33	115	85	endothermic dehydration process
	120	197	161	21.7	decomposition	137	191	155	endothermic process of disruption of interactions between MMC and β-CD
	295	387	344	27.3		241	566	439	exothermic process of disruption of interactions between MMC and β-CD
	399	591	502	38.5		570	700	654	endothermic process of β-CD
MM (2:1)	53	97	78	10.0	dehydration	28	118	78	endothermic dehydration process
	124	220	161	32.0	decomposition	133	171	155	endothermic process of MMC
	316	573	339	57.0		228	594	408	exothermic process of disruption of interactions between MMC and β-CD
			408						
FL (1:1)	28	92	74,5	12.5	dehydration	28	112	78	endothermic dehydration process
	126	197	157	21.5	decomposition	139	185	158	endothermic decomposition of MMC
	285	402	347	36.0		227	663	380	exothermic process of disruption of interactions between MMC and β-CD
	465	635	538	28.9				514	
FL (2:1)	28	86	68	10.0	dehydration	28	112	74	endothermic dehydration process
	117	205	169	30.0					
	301	388	345	21.1	decomposition	181	600	573	exothermic processes of disruption of interactions between MMC and β-CD
	465	594	540	38.9				526	

There is a great deal of similarity in the TG/DTG curves for MMC@β-CD (FL) and (MMC)_2_@β-CD (FL), in which both complexes presented four mass loss events. The dehydration event was observed at approximately 28–90 °C with mass losses of 10 and 12%, respectively, a temperature range well below that observed in free β-CD but close to that observed in free MMC, with less loss of mass. It can also be seen that the interaction between MMC and β-CD caused the decomposition of complexes (1:1 and 2:1) to occur differently from free compounds, mainly in the exothermic process of decomposition in two stages due to the two peaks, after the temperature of 187 °C, which represent the process of breaking the bonds followed by the decomposition of each material.

Anyway, the thermal analysis curves of MMs and FLs (Figure 4) have a very similar profile, but they do not overlap, suggesting that there is a difference in intermolecular interactions between MM and FL complexes. However, the influence of the guest molecule on the thermogravimetric parameters of the β-CD may indicate complexation, and observing whether the degradation of the guest molecule occurs at higher temperatures can indicate greater thermal stability of the guest molecule due to inclusion in the β-CD.^57,59^

### 3.6 Solid-State ^13^C NMR

The solid-state ^13^C CP/MAS NMR spectra recorded for the (DMC)_2_@β-CD (FL) and (MMC)_2_@β-CD (FL) inclusion complexes are shown in Figure S4; for comparison, the spectra obtained for the pure materials (β-CD, DMC, and MMC) are also included in the figures. The spectrum of β-CD exhibits a set of narrow signals at the chemical shifts expected for the carbon atoms in the d-glucopyranose units, *i.e.*, C-1: 101–104 ppm; C-4: 78–84 ppm; C-2, C-3, C-5: 71–76 ppm; C-6: 59–64 ppm. The existence of several narrow signals for each atomic site is typical of a CD sample with high crystallinity, with conformational effects accounting for the signal multiplicity.^60,61^

The ^13^C NMR spectra of DMC (Figure S4A–C) and MMC (Figure S4D–F) are also composed of narrow signals, consistent with the crystalline nature of these pure compounds. The chemical shifts of the numerous detected signals can be interpreted considering previous assignments reported in experimental and computational investigations dealing with similar types of coumarins.^62,63^ In both cases, aromatic carbon atoms not bonded to oxygen are responsible for the signals in the range of 100–145 ppm, whereas oxygen-bonded aromatic carbons give rise to signals in the range of 145–160 ppm, and the signal close to 163 ppm is due to the C=O moiety. In the case of DMC (Figure S4A–C), the signals of the two methyl groups are detected at 18.2 and 21.5 ppm, whereas for MMC (Figure S4D–F), the methyl and the methoxyl signals are detected at 18.1 and 58.7 ppm, respectively.

In the spectra obtained for the coumarin/β-CD inclusion complexes, a significant broadening is observed in all signals due to β-CD, with the disappearance of the previously mentioned signal splittings. These effects point to a reduction in the degree of crystallinity of the host and can be considered an indication of the effective formation of inclusion complexes, similar to what has been reported in previous investigations involving other CD-based complexes.^61,64^

The chemical shift region corresponding to the methyl resonances in the ^13^C NMR spectrum obtained for the (DMC)_2_@β-CD (FL) inclusion complex is shown in detail in Figure S4B. The most significant feature in this spectrum is the detection of a new signal at 19.5 ppm, somewhat midway between the two methyl signals observed for pure DMC. Also, these signals are broader in the ^13^C NMR spectrum of the inclusion complex in comparison to the corresponding signals in the DMC spectrum. These spectral changes suggest that the inclusion of the DMC molecule within the β-CD cavity affects the chemical environments of the methyl groups in the molecule, thus causing a clearly observable change in the corresponding chemical shifts. Other similar changes are observed in the chemical shift region associated with aromatic carbons when comparing the spectra obtained for pure DMC and for the (DMC)_2_@β-CD (FL) inclusion complex, as shown in the expanded view exhibited in Figure S4C. These results thus constitute further evidence for the formation of a true inclusion compound in this case.

On the other hand, the spectral changes observed in the comparison between the ^13^C NMR spectra recorded for MMC and for the (MMC)_2_@β-CD (FL) inclusion complex are much less evident, as shown in Figure S4D–F. This suggests that the intermolecular interactions between the guest molecule and the host in this case are not strong enough to cause a significant alteration in the chemical environments of the moieties responsible for the resonances detected in the ^13^C NMR spectra.

### 3.7 *In Vivo* Wound Healing Studies

There are few works related to the use of coumarins for *in vivo* wound healing studies; specifically, DMC and MMC have not been reported. Coumarins can be obtained from more than 20 different plant families or by a synthetic route and have a varied anti-inflammatory mechanism.^65^ Coumarin, 7,8-dihydroxylated, was capable of capturing radicals of activated phagocytic neutrophils that are involved in the inflammatory process.^66^ Additionally, auraptene, a citrus coumarin derivative, proved to be an effective agent to attenuate the biochemical responsiveness of inflammatory leukocytes, which may be essential for a greater understanding of the action mechanism that underlies its inhibition of inflammation-associated carcinogenesis.^67^

**Figure 5:**
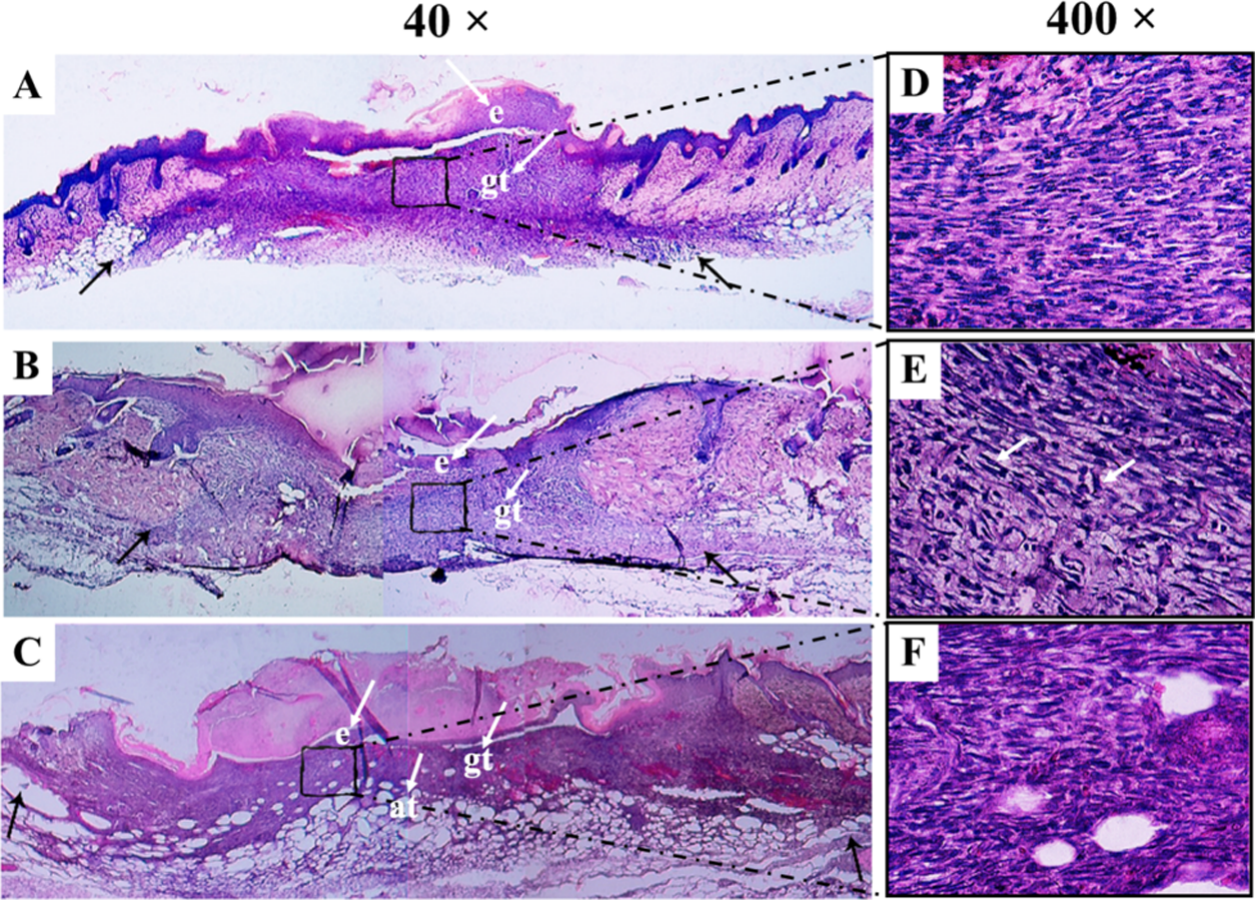
Topical application of (DMC)_2_@β-CD (FL) for 5 days after excisional injury reduces granulation tissue in the wound bed. Male mice between 8 and 10 weeks of age of the Swiss lineage had an excisional lesion of 7.0 mm diameter on the median sides of the back and received (A) 20 μL of saline solution, (B) 20 μL of (DMC)_2_@β-CD (FL) at 1 mg mL^–1^, and (C) (MMC)_2_@β-CD (FL) solution at 1 mg mL^–1^ for five consecutive days in an interval of 24 h. (A–C) Panoramic view of injured skin and the granulation tissue area stained with H&E 7 days after skin injury in mice. On day 7, postwounding re-epithelialization occurred in all groups (A–C) and a small number of fibroblasts (elongated cells) and inflammatory cells (rounded cells) was present in wounds from (MMC)_2_@β-CD (FL)-treated mice, as can be seen in the entire magnified image (E). The black arrows indicate the granulation tissue area in the wound bed, and small letters (indicated by white arrows) represent the following: e, epidermis; gt, granulation tissue; at, adipose tissue. (A–C) (40× magnification); (D–F) (400× magnification).

A topical application of (DMC)_2_@β-CD (FL) for five consecutive days following injury demonstrated an improvement in granulation tissues 7 days after the injury when compared to the saline solution (Control) and (MMC)_2_@β-CD (FL) groups (Figure 5A–C). As illustrated in Figure 5, there was a greater deposition of extracellular matrix, accompanied by a reduction in inflammatory infiltrate, a decrease in the number of fibroblasts, less crusting, and greater re-epithelialization (Figure 5B,E). These data demonstrated an improvement in wound repair. Besides, these results corroborate the better macroscopic closure of the wound 60 days after the injury, with a 12% reduction in the scar area compared to the saline group (Figure S5).

**Figure 6:**
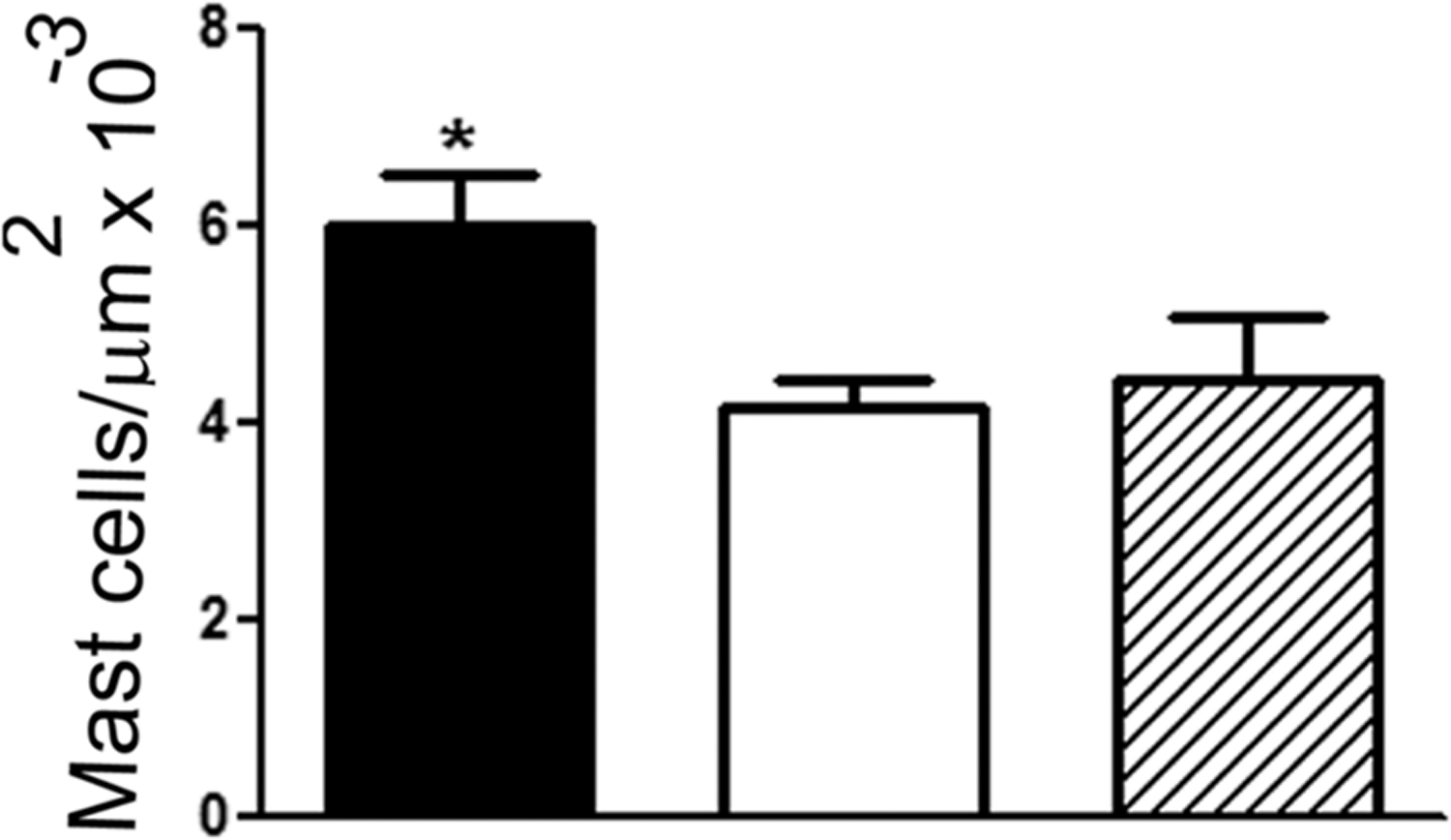
Topical application of coumarin for 5 days after excisional injury reduces the mast cell density in the wound bed. Morphometric analysis of mast cells after alcian blue safranine-stained sections. Mast cells were counted in 10 fields of 10,000 μm^2^ within the wound healing area of one section per mouse, and the results from six sections per group were expressed as the mean ± SEM saline group (black bars), (DMC)_2_@β-CD (FL) (open bars), and (MMC)_2_@β-CD (FL) (hatched bars). Data represent mean ± SEM of the mast cell density (in μm^2^ × 10^–3^). *p ≤ 0.05 compared with the saline group, *n* = 6.

Mast cells are cells that reside in the skin and are also involved in the repair process. In addition, they may be involved in allergic processes. As we are testing a new drug, it was evaluated whether these cells had changes. The groups that received topical application of (DMC)_2_@β-CD (FL) and (MMC)_2_@β-CD (FL) for five consecutive days after the injury had a reduced number of mast cells when compared to the saline group (Figure 6).

**Figure 7:**
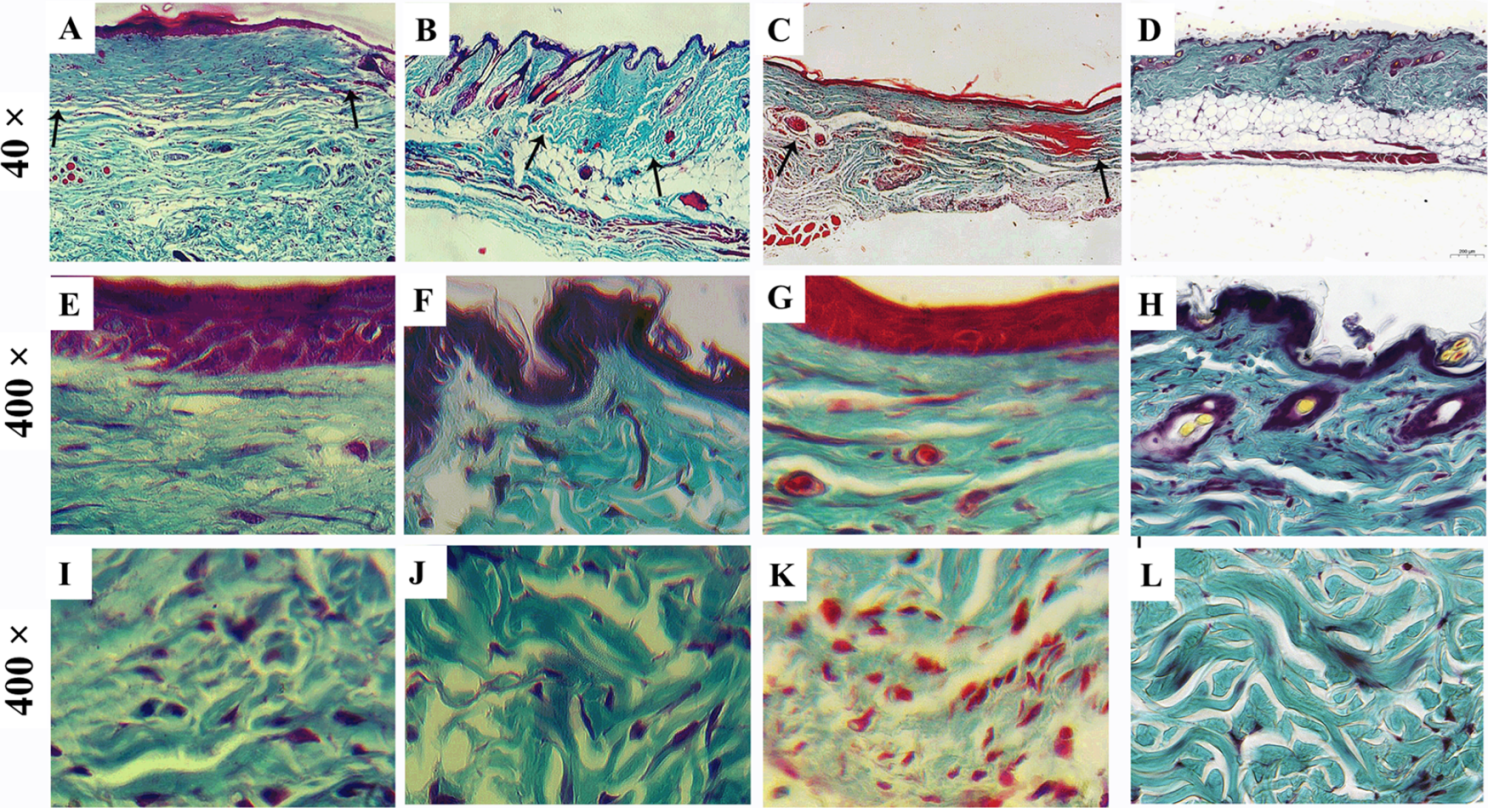
Topical application of (DMC)_2_@β-CD (FL) for 5 days after excisional injury improves collagen remodeling. Male mice between 8 and 10 weeks of age of the Swiss lineage had an excisional lesion 7.0 mm in diameter on the median sides of the back and received (A) 20 μL of saline solution; (B) 20 μL of (DMC)_2_@β-CD (FL) at 1 mg mL^–1^; and (C) (MMC)_2_@β-CD (FL) solution at 1 mg mL^–1^ for five consecutive days at an interval of 24 h. Representative photomicrographs of skin from control mice with scar tissue (A, E, I); skin of mice treated with (DMC)_2_@β-CD (FL) with scarless tissue (B, F, J); skin of mice treated with (MMC)_2_@β-CD (FL) scar tissue (C, G, K) on day 60 after wounding and normal intact skin mice (D, H, L). On day 60 after lesion, the neodermis of mice treated with (DMC)_2_@β-CD (FL) (J) closely resembles that of the normal dermis, with the collagen fibers arranged in a basket-weave pattern (L). The reorganization of the papillary dermis appears in the (DMC)_2_@β-CD (FL) group (F) at 60 days compared to intact skin (H). Arrows indicate the location of the injury. Gomori’s trichrome-stained sections. (A–D), scar area (40× magnification); (E–H), epithelium and papillary dermis (400× magnification); (I–L), reticular dermis (400× magnification).

The application of (DMC)_2_@β-CD (FL) shows a significant improvement in the repair of skin wounds in mice 60 days after the injury (Figure 7B), with a smaller scar area than that of the saline group (Figure 7) and (MMC)_2_@β-CD (FL) (Figure 7C). In addition, the group treated with (DMC)_2_@β-CD (FL) presents repair characteristics that differ from the healing process and that approximate those of the skin without injury (Figure 7H) as indicated by the formation of a papillary dermis (Figure 7F), suggested by the deposition of loose connective tissue just below the epithelium and by the formation of papillae in the epithelium that are lost in the saline control group (Figure 7E). The deposition of collagen fiber arrangements interwoven like a basketball net in the area of the reticular dermis (Figure 7J), more similar to skin without injury (Figure 7L), contrasts with the alignment of the collagen fibers typical of the scar of the saline group (Figure 7C). Treatment with (MMC)_2_@β-CD (FL) (Figure 7G,K) presents histological characteristics intermediate to the saline and (DMC)_2_@β-CD (FL). However, no dermal derivative was repaired as a hair follicle in any treatment. These data suggest that (DMC)_2_@β-CD (FL) has great potential in wound treatments.

We have demonstrated that the mice that received the topical application of (DMC)_2_@β-CD (FL) demonstrated a qualitative reduction of granulation tissue in relation to the saline group (Figure 6), probably due to the anti-inflammatory action of the coumarins as already demonstrated in other studies.^27^ In wound repairs, it has been shown that less inflammatory activity leads to better healing, more similar to the regeneration process.^9−11,68^ One of the cells that helps decrease the area of granulation tissue 7 days after the injury is mast cells, which are decreased in the groups that received a topical application of coumarins. Mast cells activate fibroblasts,^8^ and its decrease may be one of the factors that interferes with the lower presence of fibroblasts in the group treated with (DMC)_2_@β-CD (FL).

This reduction in the inflammatory infiltrate demonstrated in the topical application of (DMC)_2_@β-CD (FL) and slightly less in (MMC)_2_@β-CD (FL) interfered with the collagen deposition and remodeling phase 60 days after the injury. The animals treated with (DMC)_2_@β-CD (FL) had a smaller scar area and collagen deposition more similar to those of the intact skin. In this phase of repair, collagen III fibers are replaced by collagen I.^6^ However, no treatment led to the regeneration of hair follicles and skin appendages.

Thus, this work demonstrated that (DMC)_2_@β-CD (FL) and (MMC)_2_@β-CD (FL) reduced the inflammatory process and changed the kinetics of skin lesion closure in mice. Therefore, inclusion complexes, *i.e.*, dissolved drug molecules, can easily partition into the skin. Thus, increasing the concentration of dissolved drug molecules through formation of water-soluble drug/CD complexes increases the number of drug molecules that are able to partition into the skin and then permeate through the skin into the receptor phase.^30^ These data suggest that the topical application of (DMC)_2_@β-CD (FL) and (MMC)_2_@β-CD (FL) has great therapeutic potential in the repair of skin wounds in experimental models, but (DMC)_2_@β-CD (FL) deserves attention because of its excellent results. Still more studies should be carried out on the coumarin activity in the injury repair process.

### 3.8 Binging Affinities of DMC and MMC with Target Proteins

Docking molecular studies to explain the therapeutic potential of DMC and MMC were performed through a reverse screening approach. Proteins associated with skin wounds were selected, and the binding patterns were analyzed based on the active site of the selected enzymes. The best MolDock score and low bound energy were attributed to the best pose of the complex ligand-protein. The docking studies showed that MAPK1 and MAPK3 proteins have the highest affinity with coumarin derivatives when compared with TNF-2, ALOX5, COX-1, and COX-2 (see Table S1). Our findings corroborate the action of coumarins as inhibitors of the mitogen-activated protein kinase (MEK), particularly MAPK1 and MAPK3 inhibitors, and point out that these are promising molecular targets for tissue regeneration.^69,70^

**Figure 8:**
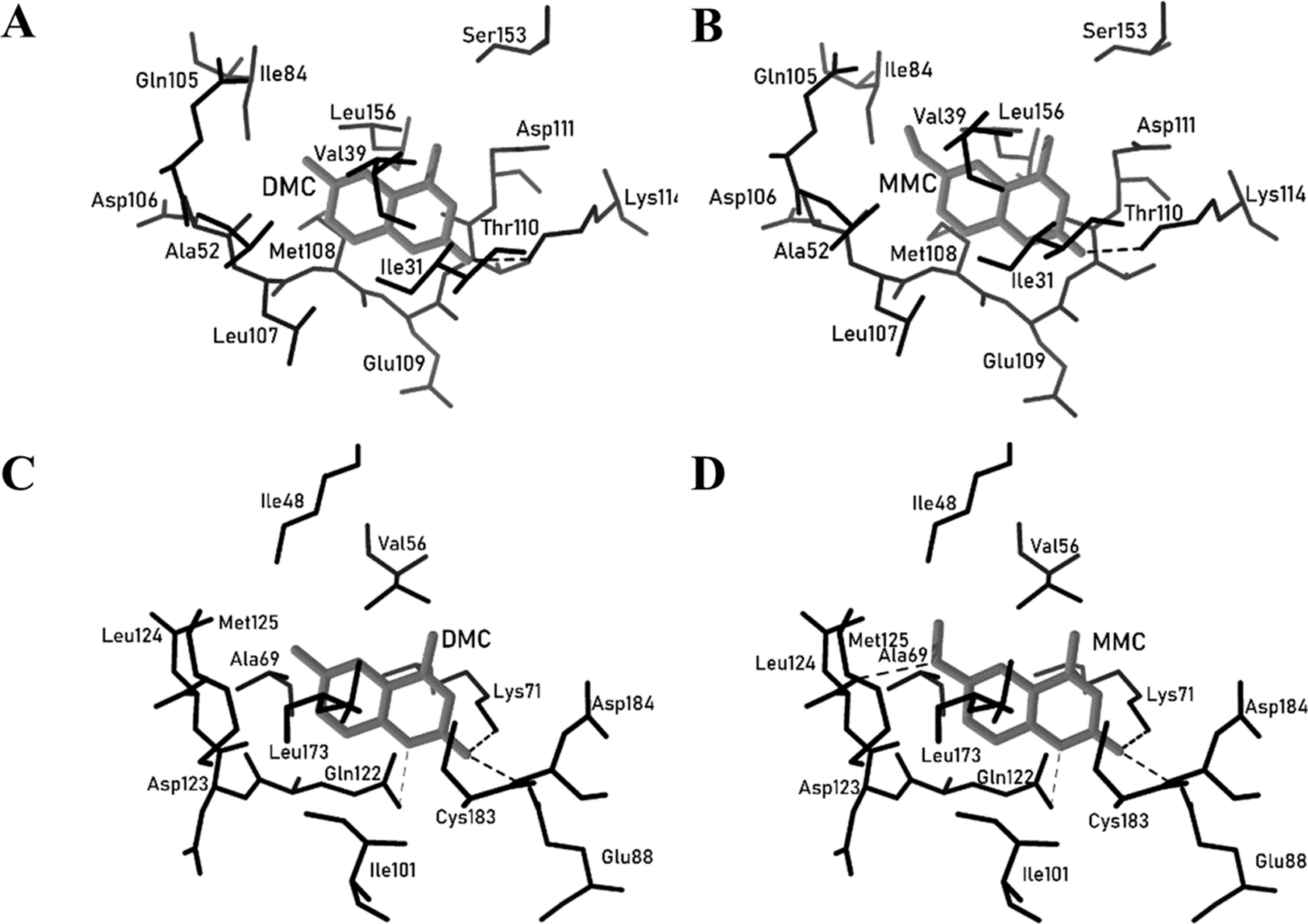
(A) DMC-MAPK1 and (B) MMC-MAPK1 complexes and (C) DMC-MAPK3 and (D) MMC-MAPK3 complexes. Dashed lines represent the hydrogen-bond interactions.

In fact, DMC formed a more stable complex with MAPK1 than MAPK3; however, the difference of the energy value is small because the structural difference between the two proteins is also small. DMC forms one hydrogen bond with the amino acid residue Lys114 (−2.41 kcal mol^–1^) of MAPK1 and three hydrogen bonds with MAPK3 (−4.68 kcal mol^–1^) (Figure 8); however, long-range electrostatic and steric (by piecewise linear potential) interactions are the main factors responsible for this difference. MMC also formed a more stable complex with MAPK1. Similar to DMC, MMC forms one hydrogen bond in amino acid residues Lys114 (−2.40 kcal mol^–1^) of MAPK1 and three hydrogen bonds with MAPK3 (−6.75 kcal mol^–1^) (Figure 8).

The complexes MMC-MAPK1 and MMC-MAPK3 were more stable than DMC-MAPK1 and DMC-MAPK3. It is possible to observe in Table S2 that the amino acid residues Gln105 and Leu156 are the main ones responsible for this variation in the MAPK1 protein. They are next to the substituted group (methyl or methoxy) in the aromatic ring. Turning now to MAPK3, the amino acid residues Asp123, Ile48, Leu124, and Met125 are the main ones responsible for the variation in the energy values (Figure 8).

## 4 Conclusions

MM and FL inclusion complexes of coumarins/β-CD were properly synthesized by a consistent and reproducible procedure, which improved the aqueous solubility of DMC and MMC against that of coumarins alone. According to the results, it was possible to conclude that there were structural differences between MMs and FLs for inclusion complexes of coumarins/β-CD. Using experimental and theoretical studies, it has been shown that the inclusion complexes are more stable at the molar ratio of 2:1 coumarin/β-CD, with hydrogen bonds and π–π stacking interactions being responsible for the enhanced stability, especially for (MMC)_2_@β-CD. The inclusion complexes were successfully characterized by TG, FTIR, solid-state ^13^C NMR, TGA, and DTA. *In vivo* wound healing studies on mice showed faster tissue regeneration in terms of re-epithelialization and enhanced collagen with the (DMC)_2_@β-CD (FL) and (MMC)_2_@β-CD (FL) inclusion complexes, clearly demonstrating that they have potential in wound healing; however, (DMC)_2_@β-CD (FL) deserves great attention because it presented excellent results, reducing the granulation tissue and mast cell density and improving collagen remodeling. The theoretical studies about binding affinities with targeted proteins suggested that these coumarin-based compounds can be MEK inhibitors, opening up a new insight for the further optimization of other MEK inhibitor scaffolds. We hope that the present study contributes to the development of a new drug composed of coumarins for a pharmaceutical formulation for the treatment of wounds and inflammatory processes, particularly for the rapid healing of diabetic skin wounds, possibly rendering it a promising therapeutic strategy for the management of diabetic wounds.

## Supporting Information Available

Additional results regarding ^1^H and ^13^C NMR spectra (400 MHz, CDCl_3_) of DMC and MMC; schematic representation of stoichiometries for the inclusion complexes; inclusion complexes for the geometries optimized; ^13^C CP/MAS NMR spectra recorded for β-CD, DMC, MMC, and for the inclusion complexes (DMC)_2_@β-CD (FL) and (MMC)_2_@β-CD (FL); scar area 60 days after lesion; and interaction energies between target proteins and studied compounds as well as interaction energy values between the main amino acid residues and proteins (PDF)
