# Supplementary material for: Coumarin/β-Cyclodextrin Inclusion Complexes Promote Acceleration and Improvement of Wound Healing
Source: ACS Appl Mater Interfaces. 2024 Jun 7;16(24):30900–14. doi: 10.1021/acsami.4c05069 (PMC11194811; doi:10.1021/acsami.4c05069)
Supplement: Supplementary file 1 — am4c05069_si_001.pdf [file am4c05069_si_001.pdf]

Supporting Information

Coumarin/β-cyclodextrin inclusion complexes reveal acceleration and improve of wound healing

Flávia Viana Avelar Dutra[1], Carla Santana Francisco[2], Bruna Carneiro Pires[1], Marcella Matos Cordeiro Borges[1], Ana Luiza Horta Torres[3], Vivian Alexandra Resende[1], Marcella Fernandes Mano Mateus[1], Daniel Fernandes Cipriano[4], Flávio Bastos Miguez[5], Jair Carlos Checon de Freitas[4], Jéssika Poliana Teixeira[6], Warley de Souza Borges[2], Luciana Guimarães[1], Elaine Fontes Ferreira da Cunha[6], Teodorico de Castro Ramalho[6], Clebio Soares Nascimento Jr[1], Frederico Barros De Sousa[5], Raquel Alves Costa[1], Valdemar Lacerda Junior[2], and Keyller Bastos Borges[1]*

[1] Departamento de Ciências Naturais, Universidade Federal de São João del-Rei, Campus Dom Bosco, Praça Dom Helvécio 74, Fábricas, 36301-160, São João del-Rei, Minas Gerais, Brazil

[2] Departamento de Química, Universidade Federal do Espírito Santo, Centro de Ciências Exatas, Avenida Fernando Ferrari, S/N, Goiabeiras, 29060-900, Vitoria, Espírito Santo, Brazil

[3] Departamento de Física, Universidade Federal do Espírito Santo, Centro de Ciências Exatas, Avenida Fernando Ferrari, S/N, Goiabeiras, 29060-900, Vitoria, Espírito Santo, Brazil

[4] Instituto de Física e Química, Universidade Federal de Itajubá, Itajubá, 37500-903, Minas Gerais, Brazil

[5] Departamento de Química, Universidade Federal de Lavras, Campus Universitário, Lavras, 37200-900, Minas Gerais, Brazil

*Corresponding author:

Prof. Keyller Bastos Borges, PhD, Departamento de Ciências Naturais, Universidade Federal de São João del-Rei, Campus Dom Bosco, Praça Dom Helvécio 74, Fábricas, 36301-160, São João del-Rei, Minas Gerais, Brazil; *e-mail: keyller@ufsj.edu.br

Figures

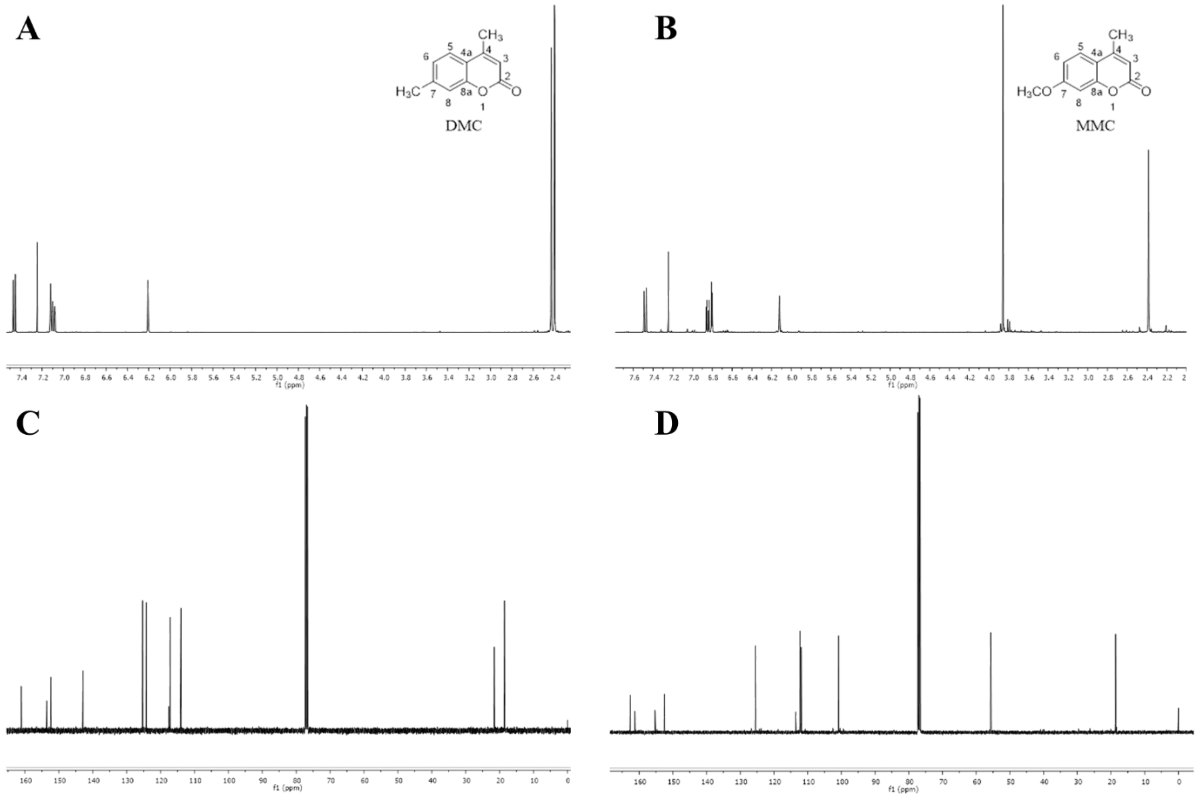

Figure S1. (**A** and **B**) ^{1}H NMR spectrum (400 MHz, $CDCl_3$) of DMC and MMC and (**C** and **D**) ^{13}C NMR spectrum (100 MHz, $CDCl_3$) of DMC and MMC, respectively. For spectral data, see section 2.1.

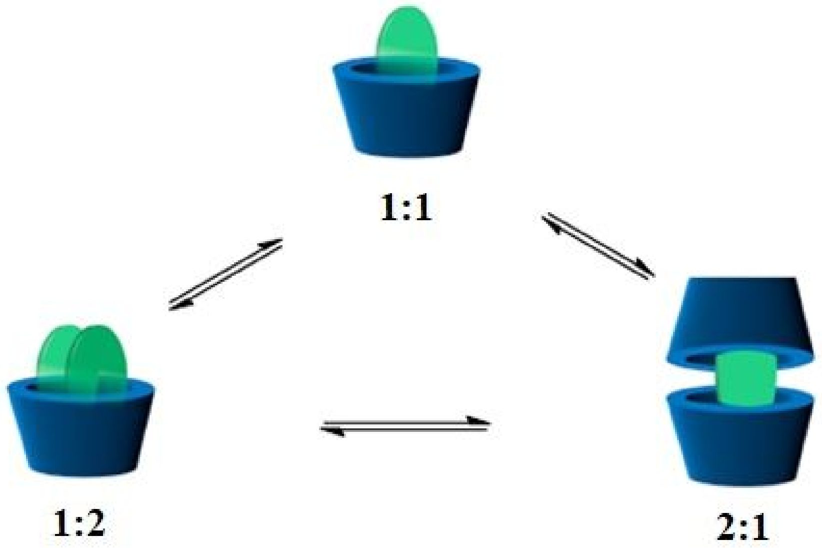

Figure S2. Schematic representation of the 1:1, 1:2 and 2:1 stoichiometries for the inclusion complexes

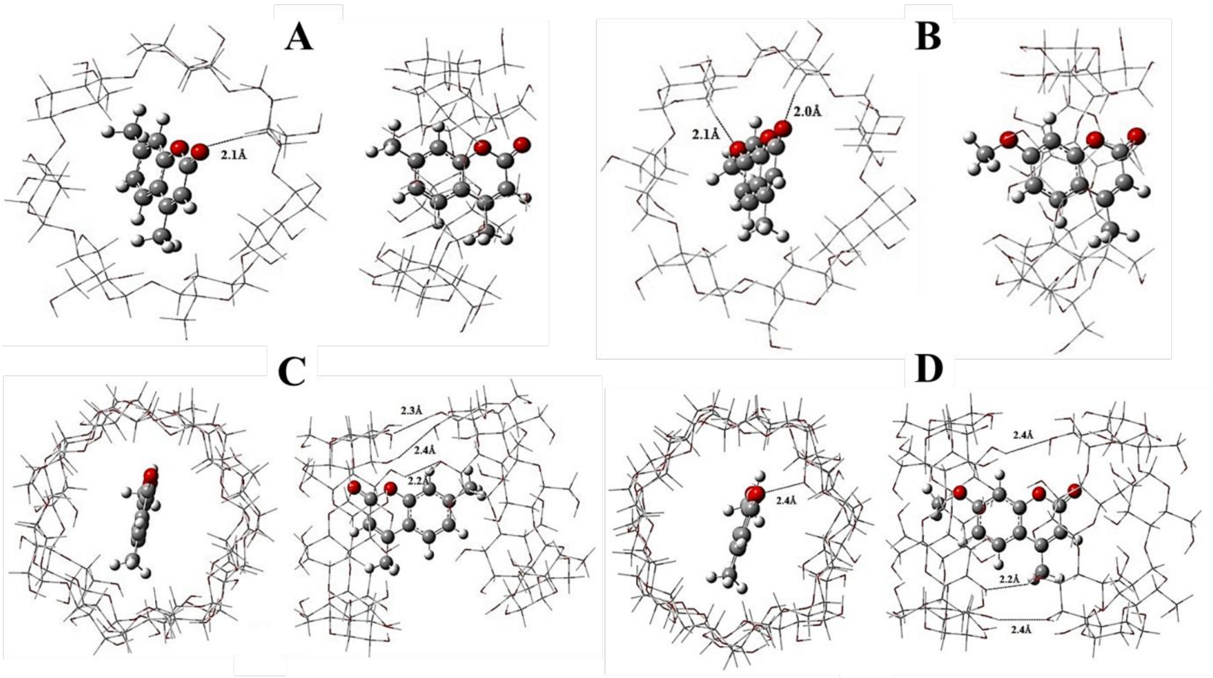

Figure S3. Inclusion complexes geometries optimized at B97D/6-31G(d,p) level of theory: **(A)** DMC@β-CD; **(B)** MMC@β-CD; **(C)** DMC@$(\beta\text{-CD})_2$ and **(D)** MMC@$(\beta\text{-CD})_2$.

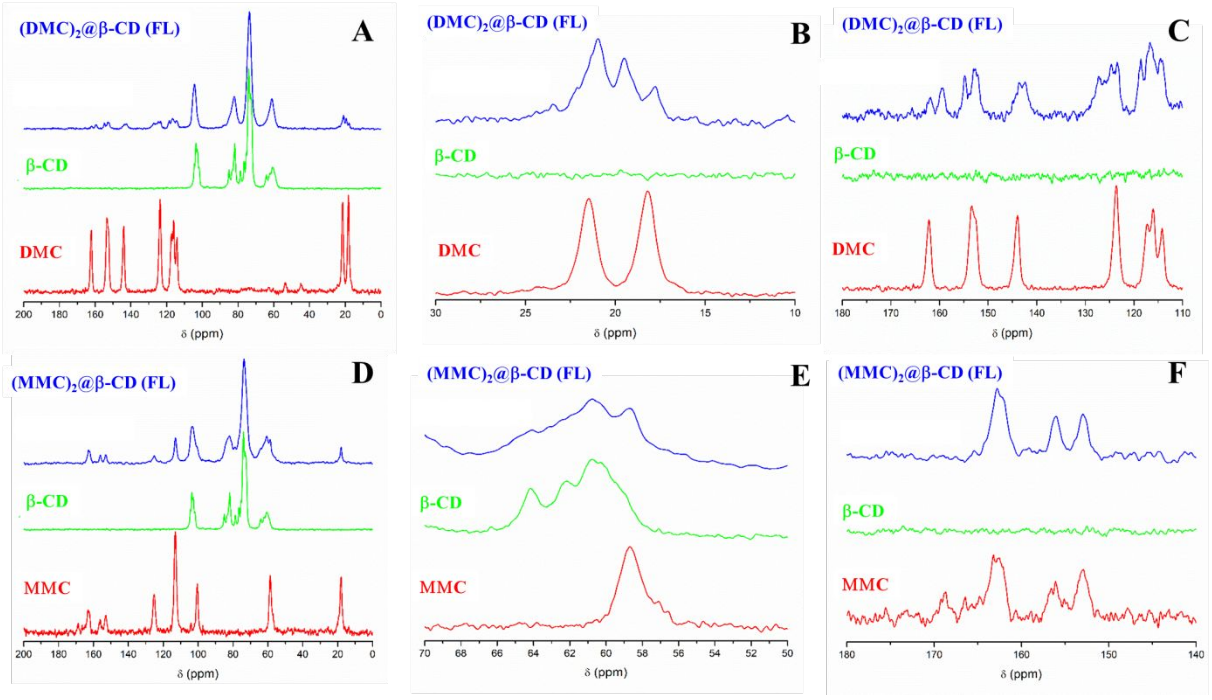

Figure S4. ^{13}C CP/MAS NMR spectra recorded for β-CD, DMC, MMC and for the inclusion complexes $(DMC)_2$@β-CD (FL) and $(MMC)_2$@β-CD (FL). The whole chemical shift range is shown in **(A)** and **(D)**, whereas expanded views around selected spectral ranges are exhibited in parts **(B)** and **(C)** for $(DMC)_2$@β-CD (FL) and **(E)** and **(F)** $(MMC)_2$@β-CD (FL).

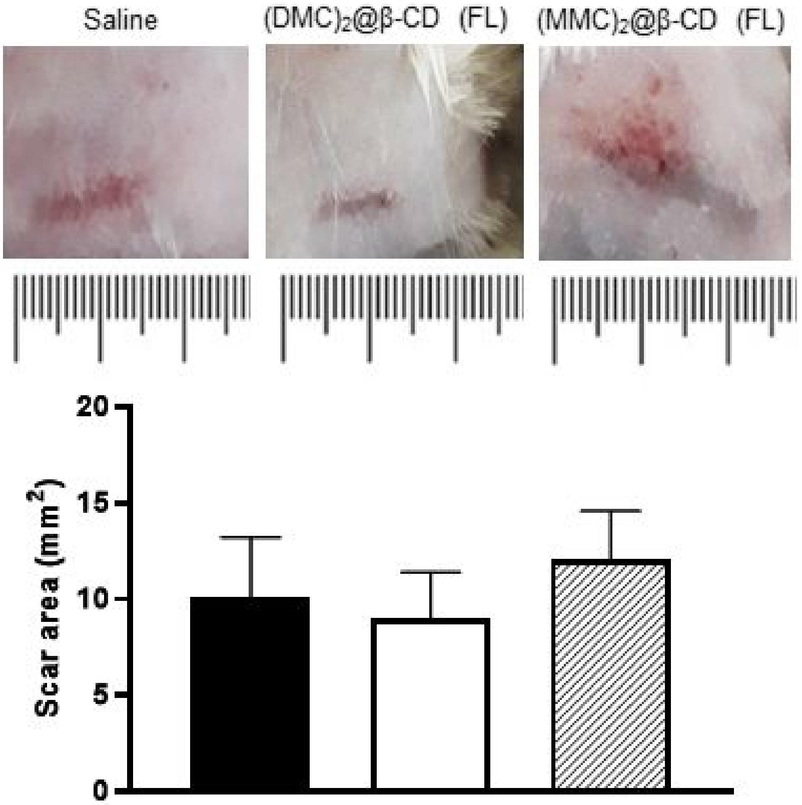

Figure S5. Scar area 60 days after lesion. (A) Scar area and (B) representative skin photographs showing macroscopic appearance of the scar, at day 60 after injuries, in saline control mice, in $(DMC)_2$@β-CD (FL) and $(MMC)_2$@β-CD (FL). Saline group (black bars), $(DMC)_2$@β-CD (FL) (open bars), and $(MMC)_2$@β-CD (FL) (hatched bars). Data represent mean ± SEM of (mm^2). $p = 0.186$, n = 6

Tables

Table S1. Interaction energies between target proteins and studied compounds.

		Moldock score energy (kcal mol^{-1})	
**Protein**	**PDB code**	**DMC**	**MMC**
**TNF-2**	**2AZ5**	-63.34	-63.35
**ALOX5**	**6N2W**	-68.40	-70.67
**COX-1**	**6Y3C**	-79.10	-83.83
**COX-2**	**5IKR**	-61.03	-73.39
**MAPK1**	**4QTA**	-89,38	-92,15
**MAPK3**	**4QTB**	-88,57	-91,29

Table S2. Interaction energy values (kcal mol^{-1}) between main amino acid residues and proteins.

MAPK1			MAPK3		
**Residue**	**DMC**	**MMC**	**Residue**	**DMC**	**MMC**
**Ala52**	-3.20	-3.86	**Ala69**	-3.29	-3.52
**Asp106**	-2.78	-2.92	**Asp123**	**-2.21**	**-1.82**
**Asp111**	-7.33	-7.28	**Asp184**	-6.16	-5.71
**Gln105**	**-1.02**	**-4.20**	**Cys183**	-8.75	-8.70
**Glu109**	-4.64	-4.59	**Gln122**	-10.18	-10.97
**Ile31**	-9.67	-9.63	**Glu88**	-0.94	-0.59
**Ile84**	-1.16	-1.87	**Ile48**	**-0.55**	**-1.20**
**Leu107**	-6.95	-6.86	**Ile101**	-6.94	-6.59
**Leu156**	**-8.78**	**-9.46**	**Leu124**	**-2.82**	**-5.69**
**Lys114**	-6.45	-6.37	**Leu173**	-10.55	-10.81
**Met108**	-10.87	-10.86	**Lys71**	-7.40	-7.53
**Ser153**	-0.52	-0.51	**Met125**	**-4.18**	**-8.91**
**Thr110**	-9.32	-9.18	**Val56**	-3.92	-3.70
**Val39**	-1.59	-2.01			
